# Some Lesions of the Pelvic Viscera Not Requiring Operative Treatment

**Published:** 1896-06

**Authors:** Bina Potter-Van Denbergh

**Affiliations:** Dansville, N. Y.


### SOME LESIONS OF THE PELVIC VISCERA NOT REQUIRING OPERATIVE TREATMENT.

By BINA POTTER-VAN DENBERGH, Dansville, N. Y.

CONSTITUTING a large part of what the busy gynecologist terms his "office work," are the nonoperative cases of pelvic disease, chronic, painful and tedious.

These cases are interesting in two lights: First, because of the prognosis, which must be reasonably frank to satisfy the patient, who, afflicted by much suffering and often by many physicians, is in a position to demand at her first consultation some statement as to the curability of her disease and the length of time necessary for the relief of its symptoms; and, secondly, because a case of this kind is sure to bring a rich reward in gratitude to the physician skilful enough to make a favorable prognosis and a restoration to health one.

In this list no mention will be made of those unnumbered cases where the various neuroses of the pelvic viscera are but reflexes from other diseased organs, when the woman is sick, not because she has a womb, but because her general health needs care and attention.

Nothing is quite so trying as the patient who insists that every ill flesh is heir to is attributable to her generative system, and whose faith and confidence are for that doctor only who yields to her opinion. These cases are legion and offer a large and remunerative field of work for the physician who permits the patient to make the diagnosis, or who fails thoroughly to investigate the sources of pain; for be it said to the honor of these cases, there is pain, often of a violent and distressing character. Many times, too, the real situation is masked by the existence of a condition of the pelvic viscera, that seems at first sight to be the source of the ill-health.

A notable case of this kind came under my care in June, 1895. The patient had slight bilateral cervical laceration, cervical erosion and endometritis.

In July I curetted the uterus, packed it with iodoform gauze and repaired the torn cervix. Patient made an excellent recovery and was slightly better of her pelvic engorgement for two months, when all her old symptoms returned in full force; the violent pelvic ache, the pain and dragging.

Here was evidently a case of double conditions. Under proper treatment directed to the liver and kidneys, with open air exercise, this patient improved and she is now enjoying her freedom from certain nervous conditions, a result due, no doubt, to the relief of pelvic irritation.

Many times these cases might yield most brilliant results under the surgeon's hands, but the patient's fear and abhorrence of an "operation" lead her to try to reach the goal of good health through any by-path rather than to endure the shorter road to freedom.

Then, again, we weigh carefully many of these cases and cannot conscientiously predict such excellent results as will encourage the patient to undergo operative relief.

Although the universal tide of opinion against wholesale oöphorectomy has possibly swung the pendulum too far, it is yet a sentiment in the right direction ; for we have not cured a diseased organ when we have cut it off or cut it out, and time has very conclusively proven that, in a large number of cases, the removal of the ovaries has entailed a far worse state of health than the condition which led the patient to consult the doctor.

Very much of the surgery of the female pelvic viscera must necessarily be experimental, for, as a noted gynecologist very recently remarked, "We are daily learning by our blunders," and when one considers the operations and the modifications of operations done upon these organs, one can but wonder when, if ever, pelvic surgery will become a finished art.

It seems a discouraging process to attempt to relieve a case of displaced and adherent tubes and ovaries by any other method than breaking up the adhesions and removing the diseased tissues, if need be. Very often the patient has fixed ideas as to the ways and means of her recovery, and it is of these cases I write.

In October, 1894, Mrs. M. consulted me, on the advice of her family physician, who refused any and all operative interference for his patient for a state of health that had interfered with walking for about three months.

At the time of consultation patient was confined to a wheel-chair. The chief seat of pain was in the left thigh and hip, in the region of the sciatic nerve. Patient had received long and vigorous treatment for sciatica, without deriving any benefit. The history of the case was simple :

Excellent health up to the time of marriage at twenty-two ; six months after marriage there were rather vague symptoms of pelvic disease, which increased for three or four years under varying forms of treatment.

Five years after marriage the patient consulted a specialist for her sterility, which was attributed to cervical stenosis; an operation (probably dilatation) was performed for its relief. Following operation was a sharp inflammatory reaction and to it the patient attributed all her subsequent ill health.

On examination the right tube and ovary were found slightly enlarged, displaced and somewhat adherent, not particularly tender. The left side of the pelvis was the chief object of interest, as it also appeared to be the chief factor of ill-health. Here the tube and ovary were evidently imbedded in a mass of exudate, as nothing could be mapped out or distinguished with the examining finger and any bimanual pressure caused such intense pain, that I was obliged to desist. Uterine body deeply engorged; bimanual, with one finger in rectum, showed it to be highly sensitive throughout, retro-displaced in second degree, not movable. Sound showed the depth to be but a trifle over the normal, endometrium deeply engorged and highly sensitive. Was obliged to give morphia to allay the pain subsequent to examination.

Fearing the possible presence of pus, I began treatment very cautiously; commencing first with vaginal applications of Churchill's tincture of iodine, followed by light tamponing with lamb's wool soaked in boro-glycerine this was followed, on its removal, by the prolonged hot douche. At the end of the first week began using the galvanic current, at first very lightly as patient seemed unable to bear a strong current, and owing to the severe pain used the positive pole in the vagina, the negative plate over the abdomen. After the fifth treatment I changed the method of application, using the negative pole over the region of the exudate in the vagina and placing the positive plate over the abdomen.

This treatment was continued for three months without interruption, gradually increasing the strength of the current as the patient could bear it up to eighty milliampères, beyond which I did not go.

In conjunction with the galvanism the patient wore the vaginal tampons of wool, dipped in boro-glycerine, had the prolonged hot douches and received daily massage, especial care being given to the affected limb.

First, improvement was noted in the ability to stand or bear her weight on left foot without much pain; then greater mobility of the limb under the hands of the masseuse, and lastly in walking.

To the bimanual examination the exudate showed gradual resolution, the uterus became more movable and the tenderness very largely disappeared.

While the patient left my care feeling that she was cured, and has subsequently led a very active life, riding a bicycle with freedom and enjoyment, she no doubt bears still the traces of long illness ; yet is perhaps stronger and is certainly happier than any operative treatment could have made her.

Though totally ignorant of the fact, this patient was the victim of gonorrhea acquired in early married life and to this fact I attributed her sterility.

My second case was a young woman aged 27, married seven years and sterile. She came to me complaining of severe dysmenorrhea, but really seeking relief in the hope of pregnancy.

On examination the uterus was found sharply anteflexed ; sound could be passed with great difficulty after drawing down the cervix firmly with a tenaculum. I found no disease or tenderness of the adnexa, nor did the patient give any history of previous pelvic disease.

Under an anesthetic, and with thorough asepsis, the uterine canal was dilated, irrigated and curetted, using the sharp curette. The cavity was then, after thorough irrigation, carefully packed with iodoform gauze and a vaginal dressing used of iodoform and boric acid.

On the fifth day after the operation the gauze was removed, having been nearly all discharged into the vagina ; vagina was thoroughly cleaned and a fresh strip of iodoform gauze carried to the fundus. This procedure was repeated twice, at intervals of three days each, when it was interrupted by the normal appearance of menstruation.

The patient made an excellent recovery, and left my care six weeks after the operation. Three weeks later her home physician wrote me that she had pelvic peritonitis, and three months after leaving the hospital she returned to my care with a well-marked case of right salpingitis, with surrounding exudate.

Dr. Mann saw this case with me in consultation and thought it the result of gonorrheal infection, but conditions could not bear out this suspicion. After the consultation this patient was at once placed on the galvanic treatment, with hot douches and ichthyol tampons.

At the end of two months the patient left me in excellent health. There were no signs left of the exudate, nor was there any pelvic tenderness.

The anteflexion had been converted into a rather marked anteversion, the sound passing readily to the fundus. This patient reports herself in excellent health, though, I deeply regret to say, still sterile.

The third and last case of this series was a patient who consulted me three months after curettement, which had been done to relieve a rather slight dysmenorrhea.

Patient single, aged 33, had been actively engaged in her profession, which was journalism. Previous health excellent.

So far as learned from her history the curettement was of the ordinary variety and without complication, recovery good. Tenth or twelfth day after the operation, following a short carriage ride, patient was seized with violent pain in the right side of pelvis extending down the thigh. Acute pain continued for several days, not very much relieved by the various forms of treatment, the limb meantime becoming flexed. Patient was placed upon local treatment consisting in the application of some medicated glycerine.

At time of the consultation the weight could be borne for a few minutes, but walking was impossible. Bimanual examination showed the right ovary enlarged, prolapsed, immovable and intensely sensitive. Left ovary prolapsed slightly to left of uterus, not sensitive. Uterus sensitive when lifted on examining finger, no discharge, depth and axis normal. The patient was very sure she would not be able to take any treatment as she was so "sore" and had already endured so much.

This case was given daily massage, gentle manipulation of the limb at first, an occasional sitz bath when the pain was particularly severe, and for a time daily, vaginal tampons of wool, saturated in ichthyol, with, of course, the daily hot douche.

At the end of four months the patient walked with ease, there was but slight pain in the hip and then only occasionally.

The pain and tenderness in the pelvis had wholly disappeared ; right ovary freely movable ; uterus no longer sensitive to bimanual manipulation, and the exudate surrounding it had disappeared. Patient was sent to the sea shore to tone up her general health and get ready for her previous occupation.

Time and patience would seem to be the moral. These are not always attainable, but in well-chosen cases proper care will usually do credit to the physician's favorable prognosis.

